# Molecular Characterization, Expression Responses and Antipathogenic Bacterial Function of Interleukin-1β (IL-1β) in Asian Seabass (*Lates calcarifer* Bloch, 1790)

**DOI:** 10.3390/biom16010046

**Published:** 2025-12-26

**Authors:** Prapansak Srisapoome, Chayanee Muangrerk, Anurak Uchuwittayakul, Ratree Wongpanya

**Affiliations:** 1 Laboratory of Aquatic Animal Health Management, Department of Aquaculture, Faculty of Fisheries, Kasetsart University, 50 Paholayothin Rd., Bangkok 10900, Thailand; chayanit.soo@ku.th (C.M.); ffisarb@ku.ac.th (A.U.); 2 Center of Excellence in Aquatic Animal Health Management, Faculty of Fisheries, Kasetsart University, 50 Paholayothin Rd., Bangkok 10900, Thailand; 3 Department of Biochemistry, Faculty of Science, Kasetsart University, 50 Ngamwongwan Road, Bangkok 10900, Thailand; fscirtw@ku.ac.th

**Keywords:** Asian seabass, *Streptococcus iniae*, *Flavobacterium covae*, interleukin-1β (IL-1β), inflammatory cytokine, antibacterial function

## Abstract

In vertebrates, the interleukin-1β molecule (IL-1β) is among the most important proinflammatory cytokines and plays crucial roles in shaping injury progression, immunological challenges, and local and systemic responses to infection. In the current study, a cDNA encoding the *IL-1β* gene in Asian seabass (*Lates calcarifer*) (*LcIL-1β*) was identified at both the nucleotide and protein levels. Its immune responses were investigated in various tissues from diseased and normal fish. Recombinant rLcIL-1β was produced in *Escherichia coli*. Furthermore, its ability to control two fish pathogenic bacteria, *Flavobacterium covae* and *Streptococcus iniae,* was assessed in vitro. Transcriptional expression was quantified by qRT–PCR, which revealed the highest levels in whole blood, followed by the liver, gills and midgut. Immune response analyses of the head kidney, whole blood, liver, gills, spleen and intestines of fish infected with *F. covae* and *S. iniae* at concentrations of 1 × 10^3^, 1 × 10^4^ and 1 × 10^5^ CFU/fish, respectively, revealed significant upregulation of *LcIL-1β* (*p* < 0.05) for 6–24 h (h) after induction. Interestingly, compared with the control treatment, the application of 1, 10 and 100 µg of rLcIL-1β greatly increased the phagocytic activity and phagocytic index of phagocytes (*p* < 0.05). Antibacterial function analyses of *F. covae* and *S. iniae* revealed minimal inhibitory concentrations (MICs) of 29.17 and 85.25 μg/mL, respectively. Finally, injection of *S. iniae* following rLcIL-1β revealed that 50 and 100 µg of the target protein demonstrated significant functional activity in safeguarding Asian seabass from these pathogenic bacteria (*p* < 0.05). This information revealed that LcIL-1β in Asian seabass significantly drives immune defense mechanisms against pathogenic bacteria, which is important for the development of effective disease prevention methods for Asian seabass aquaculture.

## 1. Introduction

Owing to its intensification, the aquaculture industry is continually hampered by disease outbreaks, which further significantly increase economic losses. Among the causative agents, various bacterial pathogens are often considered major constraints that cause serious mortality in economically important aquatic animals in aquaculture systems [1].

Similarly, the Asian seabass (*Lates calcarifer* Bloch, 1790) is classified as an economically important species in many Asian countries, including Thailand [2]. It is classified as a catadromous fish and is naturally found in subtropical and tropical areas, and it is developed to grow in various aquaculture systems, including cage or pen culture systems on the shoreline and grow-out ponds in coastal areas [2]. To date, there has been a change in farming practices from cage and earthen pond cultures near coastal shorelines to inland ponds in the eastern and central areas of Thailand, particularly Chonburi, Samut Sakhon, Chachoengsao and Samut Prakan Provinces [3], which have intensive fish farming systems. However, these areas not only face poor water quality in the culture pond environment but also experience variations in salinity from freshwater to saline environments under certain types of cultivation conditions. These phenomena notably increase fish stress and vulnerability to various harmful pathogenic bacteria, such as *Streptococcus* spp., *Flavobacterium* spp. and *Vibrio* spp [2,4]. Among these, *F. covae* and *S. iniae*, the causative agents of streptococcosis and columnaris, respectively, significantly decrease and cause severe mortality in estuarine and low-salinity culture crops within short periods, thereby increasing economic problems in the fish aquaculture industry [2,4]. Consequently, chemical treatments have been widely used by fish farmers. They effectively control target diseases in the first application. However, these treatments always raise many concerns about health risks to consumers, environmental impacts in nearby cultural areas and the ability of various fish pathogenic bacteria to develop antibiotic resistance properties [5]. Therefore, safe and highly effective control strategies are crucial for overcoming these problems.

Interleukins (ILs) are crucial cytokines that play important roles in the vertebrate immune system. Like in humans, 8 families, including 39 members, have been discovered in teleost fish [6]. Among these cytokines, the IL-1 family is crucial for innate immunity, inflammation, and immune regulation and includes proinflammatory and anti-inflammatory/antagonist members [7]. Among these cytokines, interleukin-1 beta (IL-1β) is taxonomically classified as a potent proinflammatory cytokine that belongs to the interleukin-1 family [6]. It is the most crucial proinflammatory cytokine produced by various immune cells, including the dendritic cells, macrophages and monocytes of vertebrates, to respond to the activation of toll-like receptors (TLRs) [8,9]. During NLRP3 inflammasome formation, the ligands of TLRs, cytokine receptors and NOD-like receptors (NLRs) strongly induce the expression of pro-IL-1β and NLRP3 via the myd88-NFkB pathway during the priming step [10]. In general, IL-1β is synthesized by inflammasome-mediated caspase activation to drive various immune responses [11], which are crucial for ensuring antibacterial and antiviral defense [12,13], via the interaction of IL-1β-IL-1 receptor binding. This complex can induce a phosphorylation mechanism to IκB kinase and activate mitogen-activated protein kinase (MAPK) and further drive nuclear factor kappa B (NF-kB) transcription factors and activator protein 1 (AP-1), resulting in the induction of various immune-related molecules, including small proinflammatory substances, cell adhesion molecules, acute phase proteins and chemokines [14].

Many IL-1β molecules have been reported in several teleost fish species [15]. The antiviral activity of this molecule has been investigated in orange-spotted grouper (*Epinephelus coioides*) and red seabream (*Pagus major*) [16]; however, its biological functions in fish are not well understood.

To overcome the disease problems in Asian seabass, understanding the biological functions and molecular characterization of potential immune-related genes in this economically important fish is crucial for the development of effective prophylactic and therapeutic strategies. However, this crucial information about Asian seabass is relatively limited. Therefore, the major purpose of the current research was to identify the molecular structure of the *LcIL-1β* gene. Furthermore, its mRNA expression levels have been extensively investigated in various organs of experimental fish infected with *S. iniae* and *F. covae*. The biological functions of LcIL-1β, which is produced by a prokaryotic system, against *S. iniae* and *F. covae* were analyzed by determining its inhibitory activity in both in vitro and in vivo experiments. The information from this study is crucial for developing prevention and treatment procedures against harmful pathogenic bacteria in Asian seabass aquaculture.

## 2. Materials and Methods

### 2.1. Experimental Asian Seabass

One thousand fingerling Asian seabass samples (11.4 ± 2.5 g) were obtained from a pregrow-out fish farm in Chachoengsao Province, Thailand. The experimental fish were cautiously acclimatized at the Center of Excellence in Aquatic Animal Health Management (CE-AAHM), Faculty of Fisheries, Kasetsart University, Bangkok, Thailand. Five hundred transported fish were further divided into 2,5000-L fiberglass tanks containing dechlorinated 5 ppt seawater with a fully aerated system, and the temperature was controlled at 28–30 °C. After the fish were well adapted to the laboratory conditions for 2 days, Asian seabass diet C-5003 (Uni-President, Ho Chi Minh, Vietnam) was used to raise them to 5% body weight, which was divided into 2 feeding times at 9.00 a.m. and 16.00 p.m. The fish were further quarantined for 7 days, and 5 fish from each tank were randomized to check their health using routine microbiological techniques such as agar plate streaking methods for bacterial contamination and PCR-based methods involving specific primers for contamination with 3 major viral pathogens, namely, nervous necrosis virus (NNV), scale drop disease virus (SDDV) and infectious spleen and kidney necrosis virus (ISKNV), as described previously [17].

### 2.2. Identification of the Full-Length cDNA Encoding the LcIL-1β Molecule of Asian Seabass

The head kidney was dissected from a eugenol-euthanized fish, and total RNA was further isolated using TRIzol reagent (Invitrogen, Waltham, MA, USA) in strict accordance with the manufacturer’s instructions. The resulting RNA was reverse transcribed carefully to generate 1st-strand cDNA using ReverTra Ace^TM^ qPCR RT Master Mix with gDNA Remover (TOYOBO Bio-Technology, Co., Ltd., Osaka, Japan), which contained DNase to completely destroy the genomic DNA. The primers specific to the *LcIL-1β* gene were designed and used to amplify the sequence encoding the mature protein of the *LcIL-1β* gene (LcIL-1β F and LcIL-1β R; Table 1). The specific conditions of the PCR and cloning and sequencing techniques were in strict accordance with a previous report [17]. The resulting cDNA sequence was first compared against the LcIL-1β sequence from the GenBank database (XM_018669006.2). The structure of the cDNA and deduced amino acid sequence of Lc-IL-1β were further analyzed using the BLASTx and BLASTn programs version 2.17.0 [NCBI; http://www.ncbi.nlm.nih.gov (accessed on 19 April 2025)]. The ExPASy Molecular Biology Server [http://us.expasy.org (accessed on 19 April 2025) was used to predict the molecular weight of the target protein.

### 2.3. Analysis of Homology and Evolutionary Tree Construction

The amino acid sequence identity and similarity of LcIL-1β cDNA against previously reported IL-1β gene sequences of other vertebrates were analyzed using MatGAT version 2.01 [http://www.angelfire.com/nj2/arabidopsis/MatGAT.html (accessed on 19 April 2025)]. ClustalW version 2.0.10 [http://ebi.ac.uk/Tools/clustalw/index.html (accessed on 19 April 2025)] was used to first construct the phylogenetic tree via amino acid sequence alignment, and the tree was ultimately constructed using MEGA 11.0 software [www.megasoftware.net (accessed on 25 April 2025)] by the neighbor-joining method with 1000 bootstrap replicates.

### 2.4. Protein Structural Analysis of LcIL-1β

A homology model of the LcIL-1β protein against the other interleukin-1β proteins in vertebrates was created. The model was developed using SWISS-MODEL [http://swissmodel.expasy.org (accessed on 25 April 2025)] and verified with PROCHECK [http://www.ruppweb.org/ftp_warning.html (accessed on 25 April 2025)]. Conclusively, the model was displayed using PyMOL software version 2.6.2, which is available at https://www.pymol.org (accessed on 25 April 2025).

### 2.5. Tissue Expression Analysis of IL-1β mRNA in Normal Fish by Quantitative Reverse-Transcription Real-Time PCR (qRT–PCR)

#### 2.5.1. Total RNA Isolation and First-Strand cDNA Synthesis

Five fish randomly selected from Section 2.1 were analyzed. Whole blood from all the fish was first taken from the caudal vessel of each fish using a 1-mL precoated heparinized syringe equipped with a 23-G needle. Blood cells were carefully centrifuged at 5000× *g* for 10 min, after which the blood plasma was carefully discharged, and blood cells were eventually obtained. Additionally, eleven organs, including the brain (Br), gills (Gl), head kidney (Hk), heart (Hr), intestine (In), liver (Lv), muscle (Mc), spleen (Sp), stomach (Sm), skin (Sk), and trunk kidney (Tk), were further dissected. TRIzol reagent (Invitrogen, Waltham, MA, USA) was used to isolate total RNA following the manufacturer’s instructions. The resulting total RNA was dissolved in sterile nuclease-free distilled water and quantified as a concentration using a NanoDrop. First-strand cDNA was constructed using ReverTra Ace^TM^ qPCR RT Master Mix with gDNA Remover (TOYOBO Bio-Technology, Co., Ltd., Osaka, Japan). One microgram of total RNA isolated from each fish tissue sample was separately used as the template. All the obtained first-strand cDNAs were storedt at -20 °C until use in further experiments within 2 weeks.

#### 2.5.2. Expression Analysis via qRT–PCR

To evaluate the transcriptomic levels of the *LcIL-1β* gene, 1 μg of 1st-strand cDNA synthesized from each organ from Section 2.5.1 was subjected to the Brilliant III Ultra-Fast SYBR^®^ Green qRT–PCR Master Mix (Agilent Technologies, Inc., Santa Clara, CA, USA). This analysis was conducted using an Mx3005P real-time PCR system (Stratagene, La Jolla, CA, USA) and its analytical software version 4.0 following the manufacturer’s instructions. The final volume of each reaction (25 µL) consisted of 1.0 µL of each primer pair, including LcIL-1β qF and LcIL-1β qR (Table 1), 1.0 µL of first-strand cDNA, 12.5 µL of Brilliant III Ultra-Fast SYBR Green qRT–PCR master mix and 9.5 µL of sterile DW. The qPCR profiles started with the first denaturation step at 95 °C for 10 min, and the internal 40-qPCR cycles included denaturation at 95 °C for 30 s, annealing at 56 °C for 30 s, and extension at 72 °C for 1 min. The transcriptional level of the *β-actin* gene was simultaneously detected using the specific primers Lc-β-actin qF and Lc-β-actin qF (Table 1) and used as the internal control to normalize the results and reduce fluctuations in mRNA transcription and quality and the quantity of cDNA. To confirm primer specificity, a DNA melting curve analysis was performed. All reactions involving both *β-actin* and *LcIL-1β* gene expression were conducted in triplicate for each cDNA sample. Serial dilutions in 10-fold increments of the plasmids containing the *LcIL-1β* and *β-actin* gene fragments were conducted to generate standard curves for assessing PCR efficiency. The relative copy number of the target mRNA was calculated using the 2^−ΔΔCT^ method, and the expression ratio in the muscle was technically used as a calibrator following the protocol described by Livak and Schmittgen (2001) [18].

### 2.6. Immune Response Induction Analysis of LcIL-1β Stimulated with S. iniae and F. covae

#### 2.6.1. Preparation of Pathogenic Bacteria

*Flavobacterium covae* AQAH01 and *Streptococcus iniae* AQAH02, which were separately obtained from diseased Asian seabass and stored in the CE-AAHM. Single *F. covae* colonies were first grown on Shieh agar, and a randomized colony was subsequently added to 100 mL of Shieh medium broth and vigorously shaken in an orbital incubator shaker at 30 °C for 24 h. Both media containing intact cells were centrifuged at 5000× *g* for 5 min to collect all viable bacterial cells. The supernatant was removed, and the bacterial cells were dissolved in a sterile 0.85% NaCl solution and subjected to spectrophotometry at an optical density (OD) of 600 nm to obtain a target absorbance of 1.5, which was equal to 1 × 10^8^ CFU/mL. This bacterial suspension was used for the immersion challenge experiment, as described below. Moreover, with a similar protocol, single colonies of *S. iniae* were grown on trypticase soy agar (TSA), and one colony was added to 100 mL of trypticase soy broth (TSB) and incubated under the same conditions as described above. Consequently, the bacterial cells were centrifuged at 2300× *g* for 15 min and then dissolved in normal saline solution (NSS). An absorbance of 1.0 at an OD600 of 1 × 10^9^ CFU/mL was obtained. Afterward, bacterial concentrations of 1 × 10^2^, 1 × 10^4^ and 1 × 10^6^ CFU/mL were obtained by resuspension in NSS.

#### 2.6.2. Challenge Procedure

To establish a challenge test, 180 fingerling Asian seabass samples from Section 2.1 were used, and each group of 30 fish was placed in 6 of the 250-L fiberglass tanks. Tanks 1–3 were filled with 200 L of dechlorinated freshwater, and the fish were raised under the same acclimatization procedures described above for 7 days. Moreover, 5 ppt saline water was added to experimental tanks of the same size (4–6), which were maintained under the same conditions for 7 days. After complete acclimatization, all the fish in tanks 1–3 were transferred into 10-L aquarium glass tanks containing *F. covae* bacterial solution at 1 × 10^2^, 1 × 10^4^ and 1 × 10^6^ CFU/mL, respectively, for bacterial challenge under short exposure conditions of 30 min using a scope net. The fish were then returned to their respective tanks. In tanks 4–6, all the fish were injected intraperitoneally with 100 µL of viable *S. iniae* suspension prepared in NSS at 1 × 10^2^, 1 × 10^4^ and 1 × 10^6^ CFU/mL. After the challenge, 5 fish from each experimental tank were selected randomly at 0, 6, 12, 24, 48 and 96 h. Afterward, the gills, head kidney, intestines (mid gut), liver, spleen and whole blood of each fish were harvested for subsequent analyses.

#### 2.6.3. Total RNA Extraction, First-Strand cDNA Synthesis and qRT–PCR Analyses

Tissue samples from each organ of the fish at each time point, as described in Section 2.5.2, were used to isolate total RNA, which was further synthesized into first-strand cDNA using previously described methods. The qRT–PCR assay was conducted using first-strand cDNAs from the gills, head kidney, intestine, liver and spleen of fish at each time interval as the target templates, with the same protocol described above. However, the relative expression levels of the *LcIL-1β* and *β-actin* genes at h 0 were used as calibrators in this section.

### 2.7. Recombinant LcIL-1β Protein (rLcIL-1β) Production

#### 2.7.1. Design of Recombinant LcIL-1β DNA for Protein Overexpression

The plasmid carrying the full cDNA sequence of LcIL-1β from Section 2.2 was used as the specific substrate for a double-restriction enzyme digestion reaction using *Nde* I and *Xho* I in strict accordance with the manufacturer’s instructions (Thermo Scientific, Waltham, MA, USA). All completely cut plasmids were subjected to 2.0% agarose gel electrophoresis, and the target DNA fragments of the gene of interest were purified by a QIAquick^®^ gel extraction kit (Qiagen, Venlo, The Netherlands). The obtained target fragments were further ligated into a pET28b (+) expression vector, with an N-terminal 6× His-tag and *Xho* I/*Nde* I-cut primed conditions. The ligated components were further transformed into JM109 competent cells. Luria–Bertani (LB) agar containing kanamycin at 100 µg/mL was used to select the positively transformed bacteria by culturing at 37 °C overnight. All positive clones were further extracted to obtain plasmid DNA using a plasmid extraction kit according to the previous protocol. Afterward, the positive plasmid containing the LcIL-1β sequence was again transformed into a BL21 (DE3) *E. coli* expression strain by heat-cold shock methods. To ensure the correction of the direction and nucleotide sequence of LcIL-1β, the plasmids from the candidate positive clones were sequenced as previously described in Section 2.2.

#### 2.7.2. Protein Expression of rLcIL-1β

BL21 cells containing rLcIL-1β from the above section were added to 10 mL of LB broth supplemented with 100 µg/mL kanamycin and incubated on an orbital shaker at 30 °C for 18 h as the preculture. One microliter of preculture was then added to 10 mL of kanamycin-supplemented LB broth and further incubated under the same conditions until the absorbance of the bacterial suspension reached 0.4–0.6 at OD600. Furthermore, 1.0 mM IPTG was added to the culture medium to induce overexpression, after which the cells were grown for 24 h. During this time, 1 mL of bacterial culture was sampled at 0, 4, 12, and 24 h after IPTG induction, and the bacterial cells were centrifuged and harvested at 7500× *g* for 5 min to optimize the overexpression conditions. The concentrations of expressed recombinant proteins at different time intervals were determined using sodium dodecyl sulfate–polyacrylamide gel electrophoresis (SDS–PAGE) (Thermo Fisher Scientific, Waltham, MA, USA).

#### 2.7.3. Purification and Quantitation of rLcIL-1β

A HiTrap™ Protein A HP column (GE Healthcare, South Plainfield, NJ, USA) and an ÄKTA pure protein purification system (ÄKTA pure 25 L, GE Healthcare, South Plainfield, NJ, USA) were used to purify the induced rLcIL-1β by following the manufacturer’s instructions, with some modifications based on the properties of inclusion body proteins. Technically, each aliquot of 100 mL of bacterial solution was centrifuged at 2500× *g* for 5 min. Then, the bacterial pellets were dissolved in a 50-mL conical tube containing 10 mL of 20 mM Tris-HCl (pH 8.0), and cold sonication conditions in an ice box were used. The sonicated suspension was centrifuged strictly at 12,000× *g* for 10 min to collect all the bacterial inclusion bodies. The obtained inclusion bodies were subsequently solubilized using 15 mL of solubilization buffer consisting of 6 M urea, 20 mM Tris-HCl, 5 mM imidazole, 0.5 M NaCl, and 1 mM 2-mercaptoethanol at a pH of 8.0. The recombinant rLcIL-1β was gently eluted with 20–500 mM imidazole in elution buffer (pH 8.0) by a linear gradient procedure. Fresh Eppendorf tubes were used to collect all the protein fractions. Afterward, all the obtained proteins were further analyzed for protein concentrations using the previously mentioned SDS–PAGE method. rLcIL-1β was gradually dialyzed against a solution containing 20 mM Tris-HCl and 150 mM NaCl (pH 8.0) with 20% glycerol at 4 °C overnight to eliminate small amounts of unwanted 2-mercaptoethanol, urea, and imidazole. A Bradford protein assay was used to quantify the protein concentrations. All the obtained proteins were compared to several serial standard twofold protein dilutions of albumin at an initial protein concentration of 2 mg/mL. The protein concentration was spectrophotometrically measured at an OD of 595 nm using an iMark™ Microplate Absorbance Reader (Bio-Rad, Hercules, CA, USA).

#### 2.7.4. Western Blotting

rLcIL-1β was technically separated on a 12% SDS–PAGE gel. The rLcIL-1β on the gel was quickly transferred onto a polyvinylidene difluoride (PVDF) membrane using a specific transfer buffer on an Electrophoretic Invitrogen™ Power Blotter XL System (Invitrogen, Waltham, MA, USA) at 100 V for 15 min. The transferred PVDF membrane was thoroughly blocked using a high-performance one-step blocking solution. The membrane was further incubated with an anti-6× His tag^®^ antibody (at a 1:5000 dilution for 2 h at room temperature) (Thermo Fisher Scientific, Waltham, MA, USA). Afterward, the hybridized membranes were gently washed three times with 0.05% Tween-TST. The results of protein–protein hybridization were obtained using UltraScence Pico Ultra Western Substrate (Bio-Helix, New Taipei City, Taiwan) for approximately 1–2 min. The specific reaction bands corresponding to rLcIL-1β were detected on the PVDF membrane.

### 2.8. Biological Function of rLcIL-1β: In Vitro Analysis by Phagocytic Activity

In this section, peripheral blood leukocytes (PBLs) were isolated from the whole blood of healthy Asian seabass (approximately 800 g) to analyze their phagocytic activity by following a slightly modified protocol described previously [19]. After being well anesthetized with 100 mg/L eugenol, 3 mL of whole blood was withdrawn from the caudal vein of the fish using a precoated heparinized syringe equipped with a sterile disposable 21-G needle. Each 1 mL sample of collected whole blood was released into a 15 mL PE tube containing 2 mL of RPMI (Roswell Park Memorial Institute) 1640 medium (Caissonlabs, North Logan, UT, USA) and gently mixed using a micropipette coupled with a 1 mL-cut pipette tip. RPMI-diluted blood was carefully loaded onto the surface of 3 mL of Lymphoprep^TM^ (Serumwerk Bernburg AG, Oslo, Norway) in 15 mL PE tubes and further centrifuged using a swing rotor centrifugation machine at exactly 600× *g* at 25 °C for 30 min. The target A bands of monocytes and lymphocytes (approximately 1 mL) were collected and gently mixed with 2 mL of fresh RPMI medium. The obtained leukocyte solutions were completely centrifuged at 300× *g* at 25 °C for 15 min. Then, the supernatant was carefully discarded, and phosphate-buffered saline (PBS, pH 7.4) was used to wash and resuspend the PBLs. The cells were subsequently centrifuged three times under previously described conditions. The PBL pellets were subsequently diluted to a density of 5 × 10^6^ cells/mL. Afterward, 200 µL of the PBL solution was gently layered onto twelve cover slips, with 22 × 22 mm^2^ surface areas each, and the PBLs were gradually allowed to completely adhere to the surface of the cover glass for 2 h. During this protocol, latex beads (Sigma Aldrich, St Louis, MO, USA) were serially diluted using RPMI medium to reach a final density of 1 × 10^8^ beads/mL. Four different treatments were established for each rLcIL-1β concentration. In group 1 (control), the latex beads were incubated with RPMI 1640 medium without rLcIL-1β supplementation. In groups 2, 3 and 4, the latex beads were differently exposed to rLcIL-1β at concentrations of 1, 10 and 100 µg/mL (prepared in RPMI medium), respectively, for at least 30 min. All target treatments were performed in triplicates.

Upon adhesion completion, unattached PBLs were carefully washed three to four times with 1 mL of RPMI medium each. Afterward, 200 µL of the four sets of latex beads supplemented with the rLcIL-1β solutions prepared above at 4 concentrations were loaded onto cover glasses containing a monolayer of PBLs. All the cover slips in each treatment were stored at room temperature for approximately 1.5 h to allow the phagocytes to engulf the target latex beads. Afterward, the unattached cells and unengulfed beads were carefully washed three times with RPMI medium. All remaining attached cells were stained with Dip-Quick Staining dye (Thermo Fisher Scientific, Waltham, MA, USA). Eventually, a minimum of 200 cells were carefully observed and recorded under a microscope at 100× magnification. The phagocytic activity of the PBLs, as represented by the percent phagocytosis (PA) and the phagocytic index (PI), was calculated using the following formulas:

PA = (number of phagocytosed cells with engulfed latex beads/number of phagocytosed cells) × 100

PI = number of all engulfed latex beads/phagocytosed cells

### 2.9. Effectiveness of rLcIL-1β in Controlling Pathogenic Bacteria Revealed by the Minimum Inhibitory Concentration (MIC)

*F. covae* and *S. iniae* suspensions were prepared as described in Section 2.6.1. The MICs of rLcIL-1β for these two pathogenic bacteria were determined separately using liquid growth inhibition methods. In this section, Mueller–Hinton (MH) broth was used as the standard test medium in a transparent PE 96-well microtiter plate, a U-shaped bottom type. First, rLcIL-1β was serially diluted twofold from 100 to 0.195 µg/mL (in four replicates). Second, 100 µL of the *F. covae* and *S. iniae* suspension at a concentration of 1 × 10^5^ CFU/mL was dropped into the diluted-well plate to maintain a final volume of 200 µL in each well and a final bacterial concentration of 1 × 10^4^ CFU per well. Positive control wells containing only MH broth were established, whereas wells containing bacteria and MH broth served as negative controls. Afterward, an experimental plate was incubated at 30 °C for 24 h. Third, for the interpretation step, the lowest concentration that exhibited clear broth was assumed to represent complete inhibition conditions and was defined as the MIC value. Finally, 100 µL of the combined bacteria and MH broth in each well of each test row was pipetted and carefully dropped and further spread onto MH agar plates and then incubated under the previous test environment. To determine the inhibitory effect on the tested bacteria, the growth rate of each bacterium was determined by counting colonies within the range of 30–300 colonies and finally calculated and indicated as CFU/mL.

### 2.10. Efficacy of rLcIL-1β in Enhancing Disease Resistance Against S. iniae

In this section, only *S. iniae* was used to investigate the effectiveness of rLcIL-1β in disease prevention activity using the injection route, whereas the immersion model was not suitable for rLcIL-1β application in the *F. covae* trial. The protocol details are as follows.

#### 2.10.1. Conditions and Designs

Healthy Asian seabass from the above section were randomly chosen and placed into 12 250-L fiberglass tanks (with 10 fish each) with 200 L of 5 ppt seawater to support the experimental system of 4 treatments with 3 replicates. The conditions used in this section are similar to those described in the previous section.

#### 2.10.2. *S. iniae* Induction

After 7 days of acclimation, every fish in all the experimental tanks was injected intraperitoneally with 100 µL of a 1 × 10^6^ CFU/mL *S. iniae* suspension. The fish were released back to their tanks, and 1 h later, every fish in tanks 1–3 was injected similarly with 100 µL of 0.85% NaCl to serve as a control. All the fish in tanks 10–12, 7–9 and 4–6 were separately injected with 100 µL of 0.85% NaCl supplemented with rLcIL-1β at concentrations of 100, 50 and 10 µg/mL, respectively. All the experimental fish in every tank were reared under the same conditions as above for 14 days. The daily mortality of the fish in each tank was recorded throughout the experimental period. If moribund fish were found, their livers and spleens were collected and quickly subjected to bacterial infection analyses by streaking on TSA agar plates and incubating at 28–30 °C for 24 h to confirm *S. iniae* infection.

### 2.11. Survival Curve Investigation and Statistical Analysis

The survival curve of the fish that underwent *S. iniae* induction described in Section 2.10.2 was analyzed by the Kaplan–Meier method in SPSS software version 24.0 (IBM SPSS Statistics 24.0). Significant differences between the control and rLcIL-1β injection groups were analyzed via Student’s *t*-test.

The PI and PA values were statistically analyzed with one-way ANOVA on the basis of a completely randomized design (CRD). The relative expression ratio of the *LcIL-1β* transcript at various time intervals was analyzed by two-way ANOVA with a 3 × 6 factorial design following a CRD of 2 factors (3 bacterial concentrations and 6 time intervals). Comparisons of the means of all the parameters were performed via Duncan’s new multiple range test (DMRT) with the same previously mentioned software. A result was considered significant when *p* < 0.05 or 0.001.

## 3. Results

### 3.1. Structural Characterization of LcIL-1β cDNA and Protein

**Figure 1:**
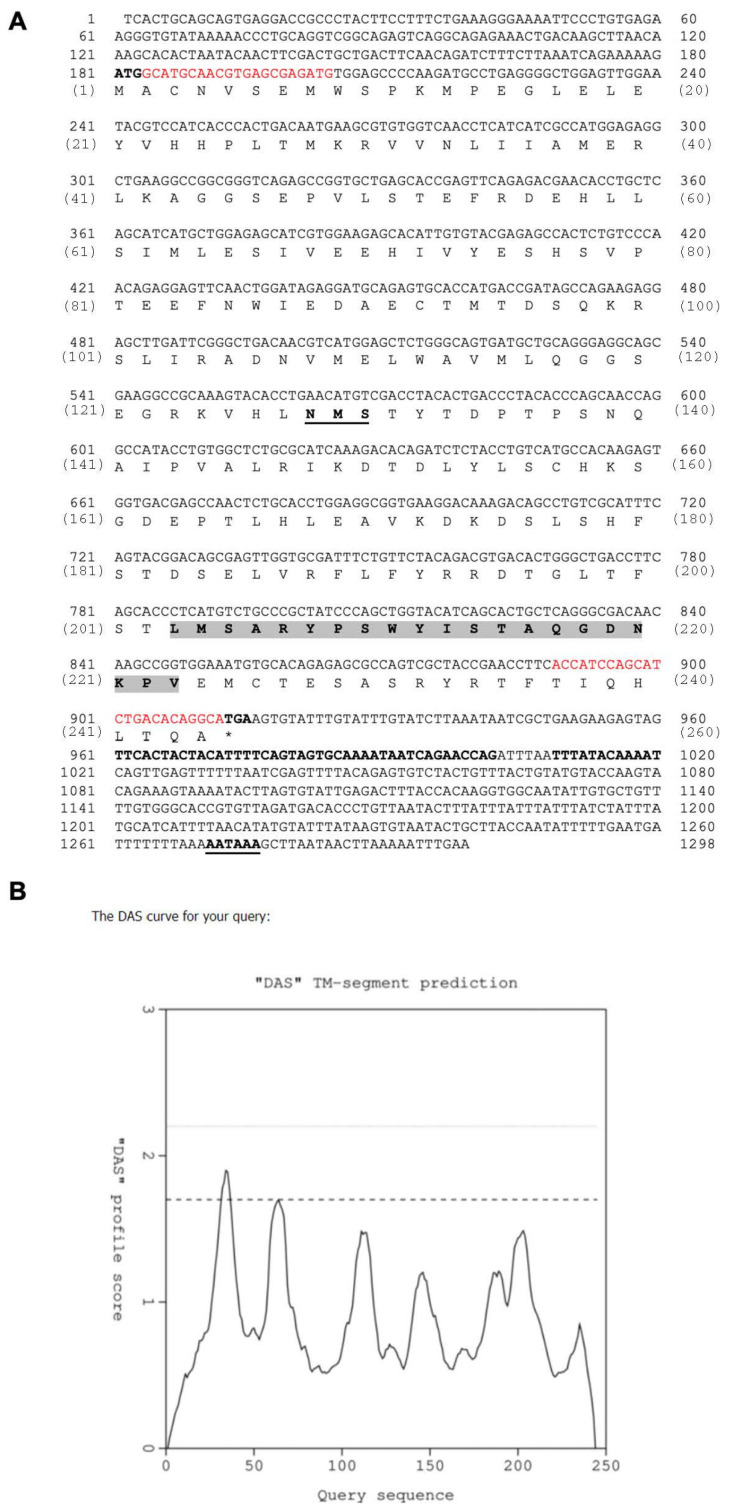
Analysis of the cDNA encoding the *LcIL-1β* gene (**A**). The upper number indicates the nucleotide sequence, and the lower number in parentheses indicates the amino acid sequence. ATG and TGA: translation start and stop codons (*), respectively; red capital letters: restriction enzyme nucleotide sequence; highlighted bold capital letters: IL-1 family signature motif; NMS: glycosylation site location; ATTTA: instability motif; and AATAAA: polyadenylation signal nucleotide sequence. DAS transmembrane prediction (**B**).

After the mature cDNA of the cloned *LcIL-1β* gene was compared with the full nucleotide sequence of the *IL-1β* gene in Asian seabass previously reported in the GenBank database (XM_018669006.2), the identity was 100%, with a total nucleotide sequence of 1298 bp (Figure 1). The predicted 5’ untranslated region (UTR) was 179 bp long and had a nucleotide sequence that could be decoded into an amino acid sequence open reading frame (ORF) of 735 bp. This sequence could be decoded into an amino acid sequence with a total length of 244 residues, a theoretical isoelectric point (p*I*) of 5.93 and a calculated molecular weight of approximately 35 kDa. The 3’ UTR was found to have a 384 bp long nucleotide sequence, consisting of one polyadenyl signal sequence (AATAAA) and a sequence of six instability motifs (ATTTA), which was detected from the 3’UTR of the *LcIL-1β* cDNA. One N-glycosylation site (NXS) was found at positions 128–130. In addition, an important amino acid sequence indicating the specific IL-1 family signature motif [FCL]-X-S-[ASLV]-X(2)-[PSR]-X(2)-[FYLIV]-[LIV]-[SCAT]-T-X(7)-[LIVMK] was found between the amino acid sequences at positions 203 and 223 at the C-terminus (Figure 1A). However, no transmembrane domain or signal peptide sequence of the LcIL-1β protein was detected (Figure 1B).

**Figure 2:**
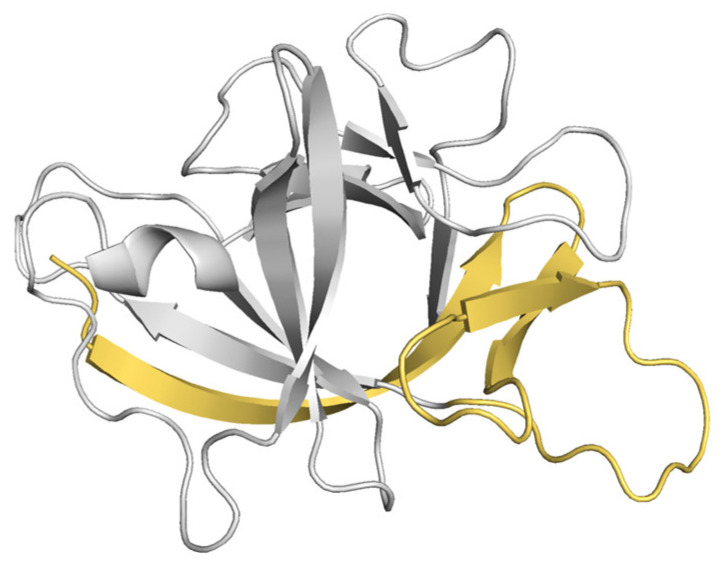
Homology model of LcIL-1β. The predicted receptor-interactive surface of the LcIL-1β model spans residues 83–125, forming a continuous patch of loops and solvent-accessible residues.

The LcIL-1β model was generated via the use of a *Gallus* interleukin-1β mutant, T117A, from PDB code 4x39.1. A, 1.61 Å resolution as a template. The modeled interleukin-1β sequence shared 32% amino acid identity with the template sequence. The homology model preserved the canonical β-trefoil tertiary structure characteristic of IL-1β family cytokines (Figure 2). Only a few residues, including A230, T199, S210, and V108, were found in the disallowed regions. These outliers are located primarily in surface-exposed or flexible loop regions, where conformational variability is expected and is less likely to affect the structural core or functional integrity of the protein. The absence of significant clustering in the disallowed regions suggests that the model is stereochemically sound and suitable for further structural and functional analyses.

**Figure 3:**
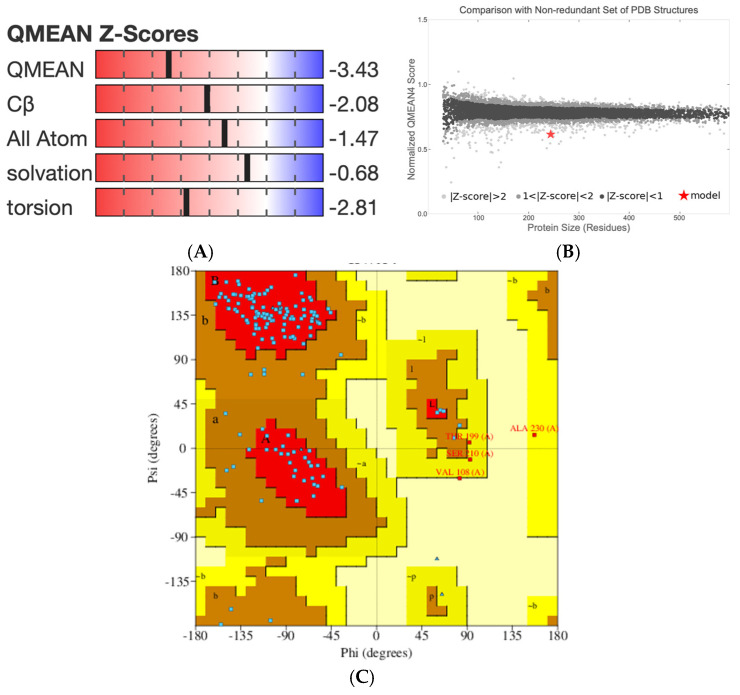
Homology model analysis of LcIL-1β. Comparison with a nonredundant set of PDB structures for the LcIL-1β model using QMEAN Z score analysis of protein size with various parameters (**A**) and normalized QMEAN scores (**B**). Ramachandran plot of the LcIL-1β homology model generated by PROCHECK (**C**).

Structural superposition with *Gallus* IL-1β (PDB ID: 4x39, chain A) revealed a conserved β-barrel core, with a root-mean-square deviation (RMSD) of 9.3 Å. The primary differences occurred in loop regions and termini, which are inherently flexible and often species- or function-specific. These regions likely account for variations in receptor interaction profiles and signaling properties. A nonredundant set of PDB structures was compared and is shown in the plot between the protein size (Figure 3A) and the normalized QMEAN score (Figure 3B). It revealed a different set of Z scores for different test parameters, including the QMEAN, C-beta interactions, all-atom interactions, solvation, and torsion. The stereochemical quality of the IL-1β homology model was evaluated using a Ramachandran plot generated by PROCHECK (Figure 3C). The plot shows that the majority of the backbone dihedral angles (φ, ψ) were distributed within the most favored regions, with significant clustering in the β-sheet (region B) and right-handed α-helix (region A) areas, which correspond to the expected β-trefoil fold of interleukin family proteins. Specifically, 85.4% of the residues occupied the most favored regions, whereas 13.9% fell within additionally allowed or generously allowed regions.

The amino acid sequence of the LcIL-1β protein was compared with those of other fish species and higher vertebrates via the CLUSTAL W program, and the amino acid sequence similarity was evaluated using the Genetyx version 7.0 program. The identity and similarity of the amino acid sequences of LcIL-1β ranged from 21.2–68.5% and 34.2–80.2%, respectively. Compared with genes from other fish species, LcIL-1β exhibited the highest amino acid identity and similarity levels with the IL-1β of Atlantic bluefin tuna (*Thunnus thynnus*), at 68.5 and 80.2%, respectively. Compared with those of avian organisms, the identity and similarity of LcIL-1β with those of mammals ranged from 24.9–29.3% and 41.1–47.0%, respectively, and between 23.4–26.9% and 34.2–41.8%, respectively. Furthermore, when the LcIL-1β gene was compared with that of reptiles and amphibians, the similarity and homology of IL-1β were found to be between 24.2–24.3% and 36.7–40.6% and between 23.5–24.1% and 40.3–41.5%, respectively (Supplementary Material Table S1).

### 3.2. Phylogenetic Tree Comparative Analysis of LcIL-1β and Various IL-1β Genes in Vertebrates

**Figure 4:**
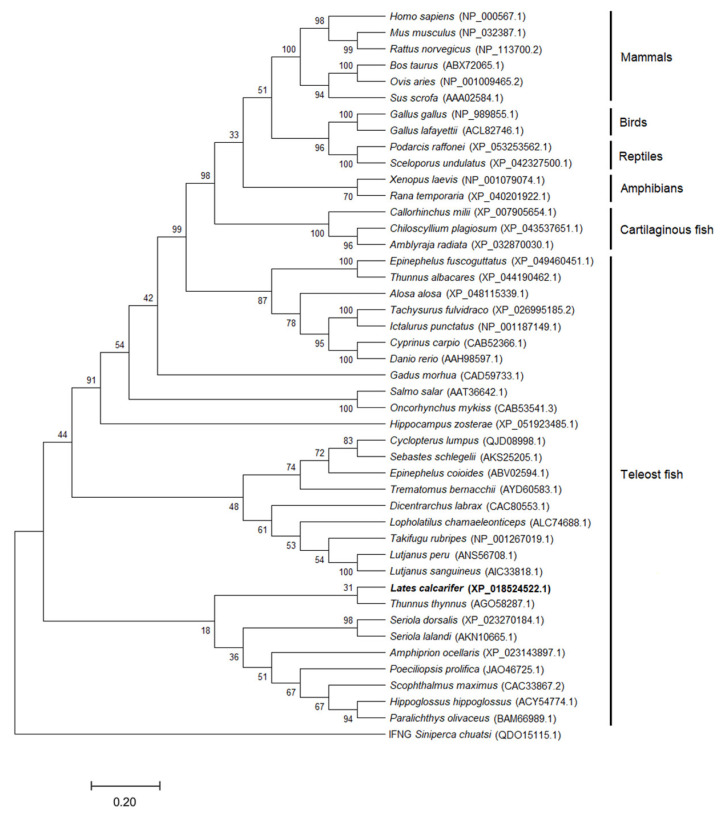
Phylogenetic evolutionary tree of the IL-1β of LcIL-1β and various IL-1bs in vertebrates. The scientific names of each species are indicated, and their accession numbers in the GenBank database are in parentheses. Interferon gamma (IFNγ) from *Siniperca chuatsi* was used as an outgroup.

The amino acid sequence relationships between LcIL-1β and IL-1β in other organisms were studied by creating a neighbor-joining phylogenetic tree to determine the evolutionary closeness of the *IL-1β* gene in various organisms, with 44 species of various organisms reported in the GenBank database, for a total of 44 species, namely, 6 mammals, 2 birds, 2 reptiles, 2 amphibians, and 32 fish species. The results of the study on the relationships between these organisms revealed that the relationships between mammals (Clade 1), birds and reptiles (Clade 2), amphibians (Clade 3), cartilaginous fishes (Clade 4), and bony fishes (Clade 5) comprised three subclades. LcIL-1β was placed in the final bony fish clade and showed a close evolutionary relationship with Atlantic bluefin tuna (*Thunnus thynnus*), with a bootstrap value of 31% (Figure 4).

### 3.3. Transcriptional-Level Analysis of the LcIL-1β Gene in 12 Tissues from 4 Healthy Asian Seabass

**Figure 5:**
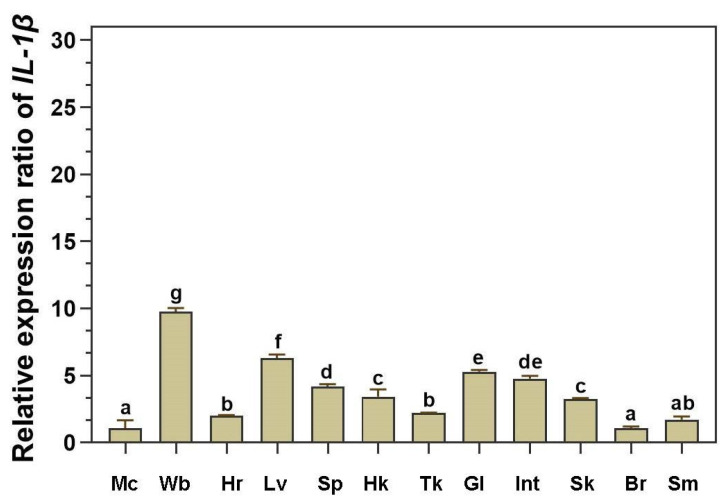
Expression pattern levels of the *LcIL-1β* gene in 12 tissues of 4 healthy Asian seabass. The mean values ± standard deviations denoted by lowercase letters are significantly different (*p* < 0.05). Abbreviations: Br; brain, Gl; gills, Hk; head kidney, Hr; heart, Int; intestine, Lv; liver, Mc; muscle, Sk; skin, Sm; stomach, Sp; spleen, Tk; trunk kidney, Wb; whole blood.

qRT–PCR was performed to determine the expression levels of the *LcIL-1β* gene in 12 tissues from 4 healthy Asian seabass. The results revealed that *LcIL-1β* gene expression clearly occurred in all the examined tissues, with the highest expression in whole blood, which was 9.78 ± 0.25-fold higher than that in muscle. Middling expression was also detected in the liver, spleen, gills, head kidney, intestines, and skin (6.30 ± 0.27, 4.18 ± 0.18, 3.41 ± 0.55, 5.28 ± 0.13, 4.76 ± 0.21, and 3.23 ± 0.08, respectively), whereas in the muscle, heart, hind kidney, brain, and stomach, *LcIL-1β* gene expression was relatively low (Figure 5).

### 3.4. Transcriptional Level Analysis of the LcIL-1β Gene in 6 Different Tissues of Healthy Asian Seabass Induced with F. covae

**Figure 6:**
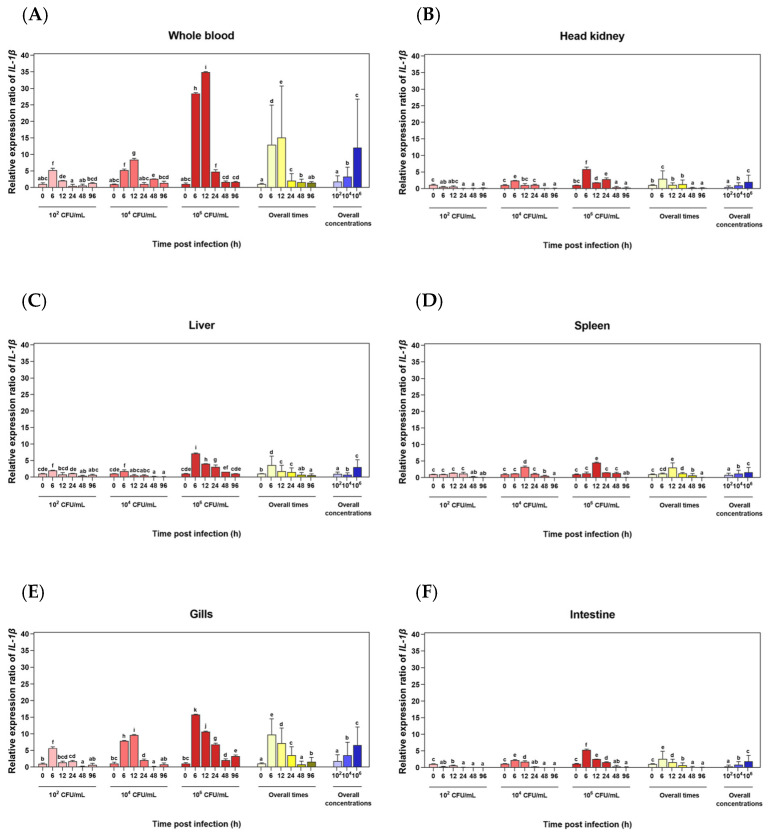
Expression analysis of the *LcIL-1β* gene by qRT–PCR in whole blood (**A**), head kidney (**B**), liver (**C**), spleen (**D**), gills (**E**), and intestines (**F**) after stimulation with three concentrations of *F. covae* at 0, 6, 12, 24, 48, and 96 h. The mean values ± standard deviations denoted by lowercase letters on each bar are significantly different (*p* < 0.05). Statistical differences were analyzed across treatments, overall times, and overall concentrations.

The results of the *LcIL-1β* gene expression in six different tissues of Asian seabass after inoculation with different *F. covae* concentrations and durations revealed that *LcIL-1β* gene expression was correlated with both the concentration and duration of inoculation. With respect to the expression of this gene in whole blood infected with 1 × 10^6^ CFU/mL *F. covae* at 6 and 12 h, the transcription levels of the *LcIL-1β* gene were greater, especially at 12 h after induction, when the transcription level of the *LcIL-1β* gene was significantly greater (34.9 ± 0.17) than at different time points in fish infected with 1 × 10^2^ and 1 × 10^4^ CFU/mL bacteria (Figure 6A). This effect was similar to that of *LcIL-1β* gene expression in the spleen (Figure 6B). At 12 h after infection with bacteria at the highest concentration, the expression level of the *LcIL-1β* gene increased to a relative expression level of 4.49 ± 0.19, which was significantly greater than that of fish infected with the lowest and moderate concentrations (*p* < 0.05). Conversely, the transcriptional levels of the *LcIL-1β* gene were significantly lower at 0, 6, 24, 48 and 96 h after inoculation. The expression levels were significantly lower at all the tested bacterial concentrations (*p* < 0.05), which was consistent with the gene expression levels when the overall time course was considered. *LcIL-1β* gene expression increased only slightly at 0 and 6 h, peaked at 12 h, and then gradually decreased to normal levels from 24 to 96 h post-infection (Figure 6D). Moreover, at 6 h after immersion at the highest bacterial concentration, *LcIL-1β* mRNA expression significantly increased in the foregut, liver, gills, and intestines, with the highest transcriptional level observed in the gill tissue, with a relative expression level of 15.8 ± 0.15 (*p* < 0.05). In addition, at 0, 12, 24, 48, and 96 h, the expression levels of the *LcIL-8* gene significantly decreased in response to all the treatments (*p* < 0.05) (Figure 6B,C,E,F), respectively.

### 3.5. Transcriptional Level Analysis of the LcIL-1β Gene in 6 Different Tissues of Healthy Asian Seabass Induced with S. iniae

**Figure 7:**
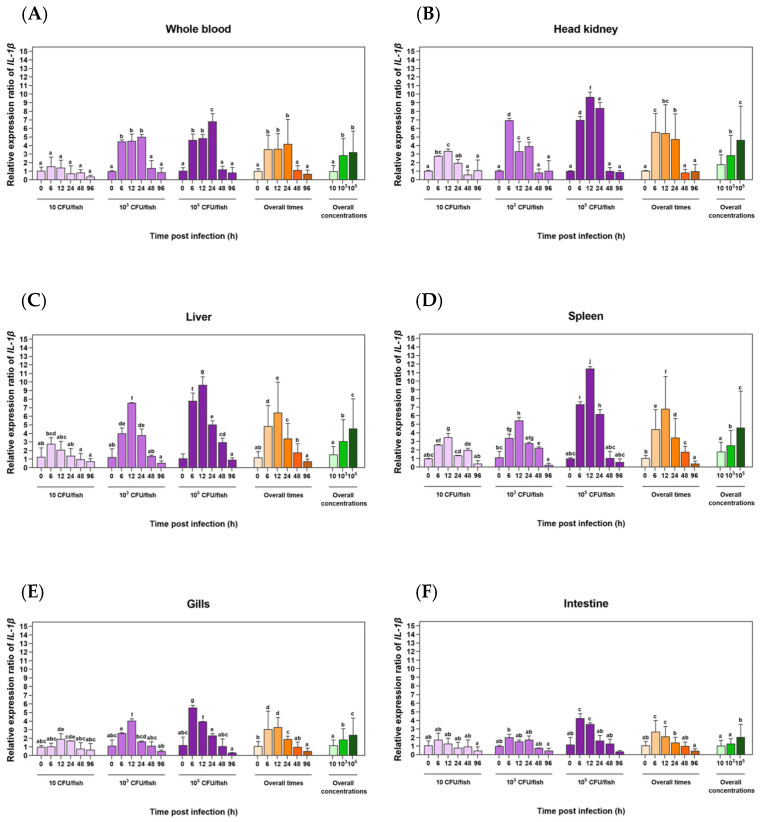
Expression analysis of the *LcIL-1β* gene by qRT–PCR in whole blood (**A**), head kidney (**B**), liver (**C**), spleen (**D**), gills (**E**), and intestines (**F**) after stimulation with three concentrations of *S. iniae* at 0, 6, 12, 24, 48, and 96 h. The mean values ± standard deviations denoted by lowercase letters on each bar are significantly different (*p* < 0.05). Statistical differences were analyzed across treatments, overall times, and overall concentrations.

*LcIL-1β* gene expression was significantly elevated in the spleen, head kidney, liver, and blood after *S. iniae* exposure at concentrations of 1 × 10^3^ and 1 × 10^5^ CFU/fish for 6 to 24 h (Figure 7A–F). In particular, at 12 h after exposure to the highest concentration, the *LcIL-1β* gene was most prominently expressed in the spleen, liver and head kidney. The relative expression ratios were 11.5 ± 0.23, 9.67 ± 0.57, and 9.65 ± 0.94, respectively (*p* < 0.05), which corresponded to the overall trend in the expression levels, indicating that the transcriptional level of the *LcIL-1β* gene clearly increased at 12 h in the fish group that received the highest concentration of bacteria (Figure 7B–D). Moreover, the overall expression time and concentration of the target genes significantly increased in whole blood at 24 h after exposure to both concentrations of *S. iniae* (1 × 10^3^ and 1 × 10^5^ CFU/fish) (Figure 7A). However, at 0, 48, and 96 h, the expression levels of the *LcIL-1β* gene significantly decreased (*p* < 0.05) at all bacterial concentrations (Figure 7A–D). A similar expression trend was also observed in the gill and intestine sections at 6 and 12 h in the fish group that received the highest concentration of bacteria. A statistically significant increase in the expression levels of the *LcIL-1β* gene was noted compared with the expression levels at different time points in fish treated with bacterial concentrations of 1 × 10^1^ and 1 × 10^3^ CFU/fish (*p* < 0.05). Conversely, the mRNA levels of *LcIL-1β* decreased continuously with increasing time and gradually decreased from 24 to 96 h across all bacterial concentrations, which is consistent with the overall time analysis showing the highest transcriptional level of the *LcIL-1β* gene at 6 to 12 h, after which it gradually decreased to baseline levels from 24 to 96 h post-infection (Figure 7E–F). Furthermore, no interaction between the pathogen concentration and time of infection was detected (*p* > 0.05).

### 3.6. Production and Purification of the rLcIL-1β Protein

**Figure 8:**
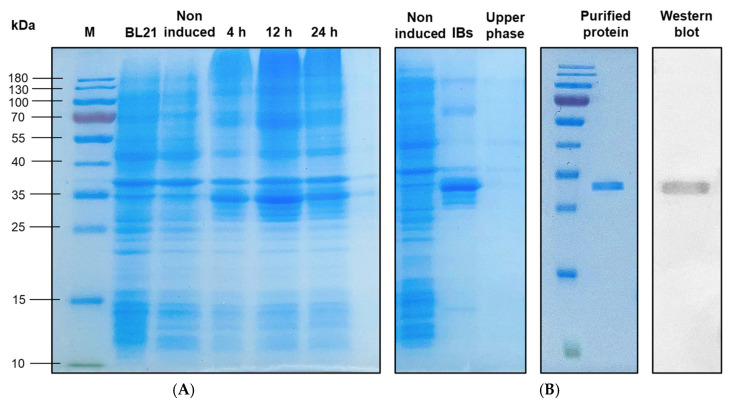
The expression of rLcIL-1β protein according to SDS–PAGE shows the appearance of recombinant rLcIL-1β proteins after different induction time intervals (**A**). M, BL21 and noninduced indicate the standard protein marker, protein from BL21 cells and uninduced BL21 cells containing pET28b(+) rLcIL-1β, respectively. Lanes 4–6 represent BL21 cells containing pET28b(+) rLcIL-1β induced with 1 mM IPTG at 4, 12, and 24 h, respectively. Expression of rLcIL-1β protein in the form of inclusion bodies (IBs) and protein purification and confirmation by Western blot analysis (**B**). The blue arrow indicates the target rLcIL-1β protein. Western blot original images can be found in the Supplementary Materials Figure S1.

rLcIL-1β was successfully produced in the *E. coli* BL21 expression system. The protein separation results based on molecular weight differences determined via SDS–PAGE revealed that after induction with 1 mM IPTG, the rLcIL-1β protein production increased after 4 h of stimulation and peaked after 12 h of stimulation. Compared with the negative control, the target protein had a predicted molecular weight of 35 kDa (Figure 8A), whose overexpression was not successfully induced by 1 mM IPTG. Afterward, when the induced proteolytic bacterial cells were lysed and the protein content was analyzed again via SDS–PAGE, rLcIL-1β protein was found to be present in the inclusion bodies compared with the upper phase. The proteins in the form of inclusion bodies were refolded before purification using an ÄKTA pure protein purification system (ÄKTA pure 25 L, GE Healthcare, NJ, USA), which revealed the emergence of only one band of the target protein. The protein expression of rLcIL-1β was subsequently determined by Western blotting. The target band of the rLcIL-1β protein was specifically bound to specific antibodies, with a protein band similar in size to that of the recombinant protein produced above, which was approximately 35 kDa (Figure 8B).

### 3.7. Biological Effects of rLcIL-1β on Phagocytosis Activity

**Figure 9:**
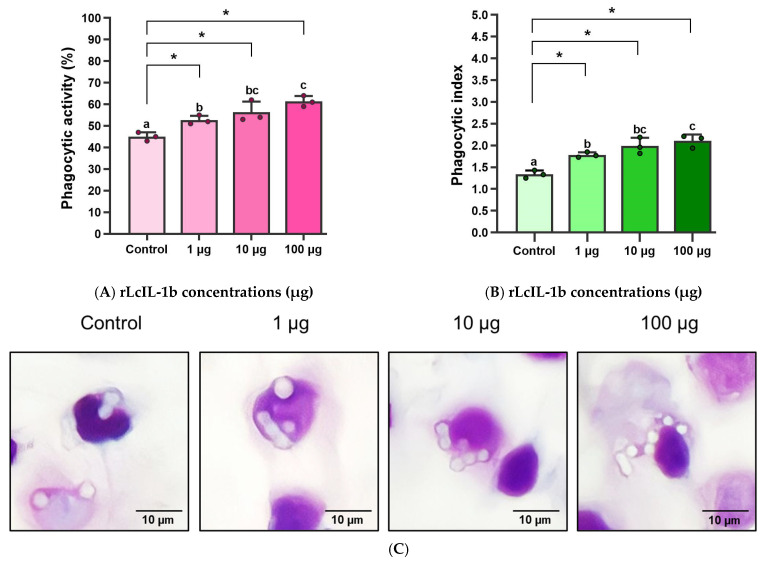
PA (%) (**A**) and PI (**B**) analyses of isolated lymphocytes incubated with rLcIL-1β protein at 1, 10, and 100 μg and an image showing the ability of leukocytes to engulf latex beads (**C**). Means ± standard deviations, denoted by lowercase letters, are significantly different (*p* < 0.05). * indicates a significant difference between control and treatments at *p* < 0.05.

The effects of different concentrations of rLcIL-1β proteins on the phagocytic activity (PA) and phagocytic index (PI) of leukocytes were evaluated. In the experimental group, in which latex beads were incubated with rLcIL-1β protein at all concentrations (1, 10, and 100 μg), the PA and PI values significantly increased (Figure 9A–C). The latex beads were ingested by white blood cells isolated from PBLs. The PA values ranged from 52.67 ± 2.08 to 61.33 ± 2.52%, which were significantly greater than those of the untreated control group (*p* < 0.05) (Figure 9A). Similarly, compared with the control treatment, treatment with rLcIL-1β at all concentrations resulted in a statistically significant improvement in the PI value (*p* < 0.05), which was 1.78 ± 0.06, 1.99 ± 0.19, and 2.11 ± 0.14, respectively, compared with that of the control, which was 1.34 ± 0.09 (Figure 9B).

### 3.8. Analyses of the Inhibitory Effects of rLcIL-1β on F. covae and S. iniae

**Figure 10:**
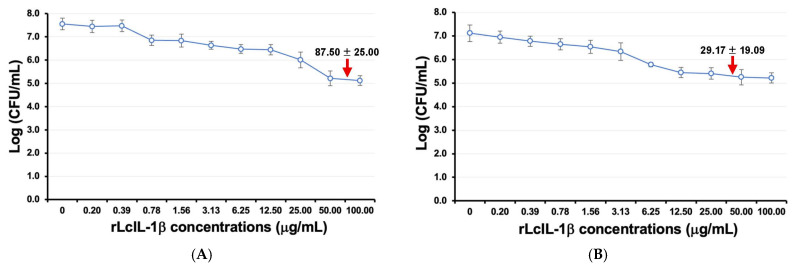
MIC concentrations reveal the biological functions of rLcIL-1β against two pathogenic bacteria, *F. covae* (**A**) and *S. iniae* (**B**). The MICs for each pathogenic bacterium are indicated by red arrows.

At concentrations of rLcIL-1β protein ranging from 50.0 to 100.00 μg/mL, *F. covae* was strongly inhibited, and compared with the lower concentrations, the solution clearly appeared after 24 h of incubation. When the experiments were performed with 4 replicates, the MIC against *F. covae* was 87.50 ± 25.00 (Figure 10A). However, rLcIL-1β protein concentrations ranging from 12.5 to 100.00 μg/mL clearly suppressed *S. iniae* growth, with an average value of 29.17 ± 19.09 μg/mL (Figure 10B).

### 3.9. Efficacy of rLcIL-1β in Preventing S. iniae Infection in Asian Seabass (In Vivo)

**Figure 11:**
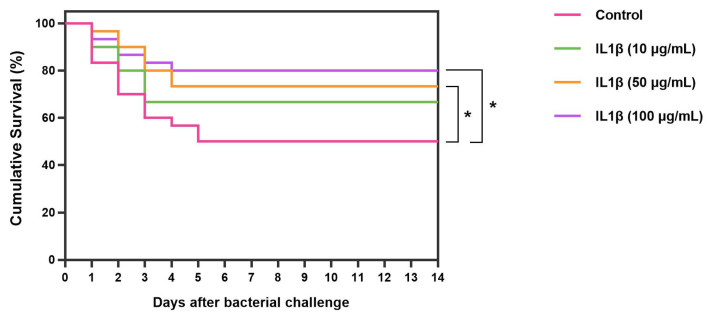
Kaplan–Meier survival curve analysis revealed the biological effects of rLcIL-1β on *S. iniae* after injection. An asterisk (*) indicates a statistically significant difference between groups of fish that were immunized with different concentrations of rLcIL-1β (*p* < 0.05).

The effectiveness of rLcIL-1β proteins on disease resistance after inoculation with the pathogenic bacterium *S. iniae* was tested. From days 4 to 14, compared with control fish, fish injected with rLcIL-1β protein at concentrations of 50 and 100 μg/mL exhibited significantly higher survival rates (73.33 ± 2.72% and 80.00 ± 1.93%, respectively), with a survival rate of 50.00 ± 5.27% (*p* < 0.05). However, during that time, the survival rates of the fish in the rLcIL-1β group treated with 10 μg/mL were lower than those of the fish in the other groups injected with higher protein concentrations, and no significant difference was found between the survival of the tested fish in the group treated with the lowest protein concentration and that in the untreated group (*p* > 0.05) (Figure 11).

## 4. Discussion

In the current study, cDNA encoding the proinflammatory cytokine LcIL-1β gene was successfully cloned and characterized in Asian seabass. The amino acid transcription component was able to decode to 244 amino acid residues, in which the ORF contained a glycosylation site, A_128_-M-S. This analysis is consistent with the study of the other IL-1β genes in several fish species, including South American fish (*Piaractus mesopotamicus*) [20], grass carp [21], Nile tilapia [22], and small spotted catshark [23]. In addition, an important structure showing the specificity of the IL-1 family signature motif [FCL]-X-S-[ASLV]-X(2)-[PSR]-X(2)-[FYLIV]-[LIV]-[SCAT]-T-X(7)-[LIVMK] was found, indicating that the *LcIL-1β* gene plays a role in the immune response similar to that of other vertebrates. In addition, a study on the position of the cleavage site of the signal peptide using the DAS Transmembrane prediction program revealed that the cDNA of the *LcIL-1β* gene did not contain such a site. These findings are consistent with reports in other fish species [24,25], suggesting that the *IL-1β* gene requires processing to release the protein into its active form [26]. In addition, six nucleotide sequences containing the ATTTA motif, also referred to as the AU-rich element motif (ARE), were detected at the 3’ UTR end. This nucleotide sequence is considered an important characteristic of inflammatory genes, such as cytokines, chemokines, and oncogenes, which generally have short half-lives because of the presence of the AU-rich element motif, which is important for mRNA stability [27,28].

An evolutionary tree was created using amino acid sequences to explain the evolutionary closeness of the *IL-1β* gene among reported organisms. Analysis of the *LcIL-1β* gene and 44 other organisms deposited in the GenBank database revealed that the relationships could be divided into 7 groups. LcIL-1β is classified into the IL-1β group of bony fish, whose evolutionary relationships are closely related to those of Atlantic bluefin. The obtained data were consistent with the comparison of the similarity and homology of the *LcIL-1β* gene with those of other organisms. The highest similarity and homology values were found for the *IL-1β* gene of Atlantic bluefin tuna, at 68.5 and 80.2%, respectively. These results indicate that the *LcIL-1β* genes are evolutionarily related and that the genetic characteristics of this gene are conserved with those of the tuna group.

Through the use of qRT–PCR techniques, the transcription of the *LcIL-1β* gene in healthy Asian seabass revealed that the *LcIL-1β* gene was ubiquitously expressed in all the tested tissues, with the highest expression in whole blood, indicating that the key components involved in both cellular and humoral immune responses, especially leukocytes, were involved. When pattern recognition receptors (PRRs) of fish are stimulated with molecules of pathogens or foreign substances entering the body, either PAMPs or MAMPs, cells involved in such immune responses respond by initially secreting IL-1β, which is among the most proinflammatory cytokines and causes the organism to immediately respond to infection, causing a chain of reactions leading to inflammation [8,29]. In addition, IL-1β can further induce the production of several chemokines in target cells at the site of infection to perform specific functions [30]. *LcIL-1β* gene expression is consistent with that of several other higher vertebrates, such as the Chinese soft-shelled turtle, in which the expression level of the *IL-1β* gene is greatest in the blood, followed by the intestines and spleen [31]. However, Jiang et al. (2008) [32] reported that the transcriptional level of the *IL-1β* gene is relatively low in the spleen, kidney, gill, and intestine of yellow seabream. Moreover, the expression of this gene was not detected in the heart, liver or muscle of seabream [33,34], whereas in Atlantic bluefin tuna, the transcriptional level of this gene was higher in the liver than in the foregut [35], suggesting that under normal conditions, *IL-1β* gene expression varies across tissues [36].

It is well known that the route of infection significantly affects the pathogen–host relationship. Therefore, discerning the relationships between different routes of infection and fish immune responses is needed. In this study, the transcriptional patterns of *LcIL-1β* in response to systemic infection with *S. iniae* and exterior infection with *F. covae* were studied using qRT–PCR in immune-related tissues of fish, namely, the liver, spleen, forepaw kidney, blood, gills, and intestines, which are the major lymphoid organs in bony fish [37]. The results of this study revealed that the *LcIL-1β* gene was most highly expressed in the fish spleen, followed by the liver and head kidney. After injection with *S. iniae* at the highest concentration at 12 h, these results are similar to those of previous reports of *IL-1β* gene expression under bacterial and/or LPS and poly(I:C) induction in Atlantic cod [38], rough skin sculpin [39] and black rockfish [36], in which the highest gene expression was detected in the spleen and fore kidney. This pattern could have occurred because the spleen is an important organ responsible for catching pathogens or foreign substances into the body. The spleen is an organ that plays a role in blood cell production. It is also like a home or school for the development of many white blood cells. It includes fully developed blood cells that migrate to the spleen, making it an important site for both cell-mediated and protein- or fluid-mediated immune responses [40]. The head kidney in bony fish is considered the main immune organ responsible for the ingestion of pathogens and foreign substances. It presents antigens to specific immune cells and serves as a site for lymphocyte production and development [41]. Furthermore, a similar expression pattern of the *LcIL-1β* gene was observed in the gills and intestines at 6 and 12 h in fish induced by the highest concentration of *S. iniae*. In the blood, increased expression levels of this gene were also observed at 24 h after exposure to both concentrations of *S. iniae*, indicating that these organs, particularly the spleen and anterior kidney, are crucial for the *S. iniae* response in Asian seabass.

Furthermore, another pathogenic bacterium that has caused significant damage to Asian seabass aquaculture in Thailand is *F. covae*. After exposure to *F. covae* at a concentration of 1 × 10^6^ CFU/mL, the *LcIL-1β* gene was clearly expressed in all the examined tissues. The highest expression level in whole blood occurred at 12 h because *F. covae* was able to induce white blood apoptosis in response to the infection site. *F. covae* also contains the chondroitin AC lyase enzyme, which strongly degrades various polysaccharides in host connective tissue [42]. Therefore, this bacterium can pass through the mucosal immune system in the gills of fish. As a result, some bacteria begin to spread, invade and multiply in the bloodstream, causing systemic infection or infection that spreads to other parts of the body [43]. In addition, the transcriptional levels of the *LcIL-1β* gene in the spleen were similar to those in whole blood. The expression level of the *LcIL-1β* gene increased at 12 h after exposure to the highest bacterial concentration. At 6 h after immersion with the highest concentration of bacteria, *LcIL-1β* expression was elevated in the gills, head kidney, liver and intestines. These observations clearly demonstrate the important role of *LcIL-1β* as a proinflammatory cytokine. However, from 24 to 96 h, the expression levels of the *LcIL-1β* gene decreased across all the bacterial concentrations to which the fish were exposed. These results suggest that the *LcIL-1β* gene has a short half-life in response to pathogens because of the presence of a nucleotide sequence known as an AU-rich element motif or ARE at the 3′ UTR end. This motif plays a crucial role in abating the reaction response by controlling the protein synthesis process [44]. It also involves the inhibitory and regulatory mechanisms of several cytokines, as their overexpression directly affects cell damage [45].

Previous reports clearly revealed that IL-1β increases phagocytic activity by promoting monocytes maturation into highly efficient macrophages, increasing the expression of key Fc receptors (CD16 and CD64) and scavenger receptors (CD36, CD163, and CD206) and enhancing the uptake of particles [7]. In this study, the rLcIL-1β protein was successfully produced and further used to induce phagocytosis in fish PBLS, and rLcIL-1β at concentrations between 1 and 100 μg was clearly sufficient to induce phagocytosis of phagocytes in vitro. This information is consistent with that of Hong et al. (2003) [46] in rainbow trout, who reported that the PA and PI values could be efficiently increased at a concentration of 1 μg of rOmIL-1β protein. However, when the protein concentration was increased to 10 μg, the PA and PI values significantly decreased (*p* < 0.05). Similarly, an investigation by Peddie et al. (2002) [47] revealed that higher IL-1β concentrations decreased the efficiency of phagocytic yeast engulfment, which is expected to be a result of differences in saturation and/or sensitivity to IL-1 receptor stimulation among fish species.

The biological functions of rLcIL-1β include inhibition of the growth of *S. iniae* and *F. covae*, as indicated by the MICs of 29.17 and 85.25 μg/mL, respectively. The concentration used to control *F. covae* was greater than that used to control *S. iniae,* indicating that the difference in the Gram type of bacteria or bacterial cell wall structure may directly affect the biological functional mechanisms through which rLcIL-1β effectively inactivates these bacteria. Additionally, *S. iniae* are spherical Gram-positive cocci with diameters of approximately 0.3–0.5 μm [48], whereas *Flavobacterium* spp., which are Gram-negative long rod bacteria that are 0.45 μm wide and 2–10 μm long [49], may require a much greater amount of rLcIL-1β to control it. Further studies are needed to clarify this possibility.

Notably, in humans, IL-1β plays a role in stimulating important cytokine molecules, namely, IL-26 and IL-8, which function as antimicrobial peptides (AMPs) [17,50] that are secreted from Th17 cells [50]. In addition, IL-1β can also induce the amniotic membrane of the developing fetus to secrete HBD-2, HBD-3 and the cathelicidins LL-37 and elafin, which have AMP properties [51], or release hepcidin and β-defensin1 in grass carp (*Ctenopharyngodon idella*), which crucially prevents *V. mimicus* by downstream activation of the NF-κB pathway [52]. Interestingly, from the initial findings of Jayaraman et al. (2013) [53], it was reported that IL-1β has direct antimicrobial activity in macrophages infected with *Mycobacterium tuberculosis*, which suggests that in addition to its function in stimulating the secretion of AMPs. In our study, we demonstrated that IL-1β itself has the potential to be an AMP. Previously, it has been found that some cytokines possess a direct, non-signaling antimicrobial function by disrupting bacterial cell membranes or interfering with essential microbial processes [54,55]. However, further studies on the antimicrobial effectiveness of IL-1β and the in-depth mechanisms of this cytokine in Asian seabass and other bony fish species should be conducted in the future.

Finally, the effectiveness of rLcIL-1β against *S. iniae* was evaluated, and 50 and 100 μg/mL rLcIL-1β protein concentrations significantly protected the fish from the target pathogenic bacterium. This information is consistent with that of previous studies that revealed the effectiveness of the rLcIL-1β protein in inhibiting the growth of *S. iniae* in vitro. These findings suggest that the rLcIL-1β protein plays an additional crucial role in shaping *S. iniae* resistance in Asian seabass because of its ability to secrete cellular and humoral factors and induce the phagocytic activity of phagocytes and antimicrobial activity itself [50,51,53], including the immune function of IL-1β in inducing the expression of acute-phase proteins by hepatocytes, which increases in the body with the occurrence of acute inflammation, further helping to destroy toxins and eliminate microorganisms. In addition, IL-1β also has powerful bactericidal activity [56,57].

## 5. Conclusions

In this study, the expression and functional analysis of the IL-1β molecule in Asian seabass were performed. Expression analysis of *LcIL-1β* under the induction of two pathogenic bacterial strains revealed early responses after induction, indicating that LcIL-1β plays a functional role in acute responses to bacterial infection in the fish immune system. Functional analysis of rLcIL-1β revealed a strong increase in phagocytosis, indicating that proinflammatory cytokines increase phagocytic activity. Interestingly, rLcIL-1β exhibited direct antibacterial activity in an in vitro system, which is the first report in Asian seabass. Additionally, rLcIL-1β significantly induced protection against *S. iniae* under in vivo conditions. The obtained information is crucial for further application in reducing severe bacterial diseases in Asian seabass aquaculture for the relief of economic losses caused by these bacterial infections.

## Figures and Tables

**Table 1:** Primers employed for overexpression of the LcIL-1β recombinant protein and qRT–PCR analysis.

Primer Code	Sequence Direction (5′–3′)	PCR Amplicon Size	Purpose
LcIL-1β F	CATATGATGGCATGCAACGTGAGCGAGATG	732 bp	Protein overexpression
LcIL-1β R	CTCGAGTGCCTGTGTCAGATGCTGGATGGT		
LcIL-1β qF	TTCAGAGACGAACACCTGCTCA	234 bp	qRT–PCR
LcIL-1β qR	GGTCGACATGTTCAGGTGTACT		
Lc-β-actin qF	TACCCCATTGAGCACGGTATTG	150 bp	qRT–PCR
Lc-β-actin qR	TCTGGGTCATCTTCTCCCTGTT		

## Data Availability

The original contributions presented in this study are included in the article/Supplementary Materials. Further inquiries can be directed to the corresponding author.

## Supplementary Materials

The following supporting information can be downloaded at https://www.mdpi.com/article/10.3390/biom16010046/s1; Table S1: Homology of the nucleotide and amino acid sequences of the *LcIL-1β* gene in Asian seabass compared with those in other vertebrates; Figure S1: The original Western blot images for Figure 8B.
