# Supplementary material for: Molecular Characterization, Expression Responses and Antipathogenic Bacterial Function of Interleukin-1β (IL-1β) in Asian Seabass (Lates calcarifer Bloch, 1790)
Source: Biomolecules. 2025 Dec 26;16(1):46. doi: 10.3390/biom16010046 (PMC12838536; doi:10.3390/biom16010046)
Supplement: Supplementary file 1 [file biomolecules-16-00046-s001.zip › biomolecules-3998479-supplementary.pptx]

## Slide 1
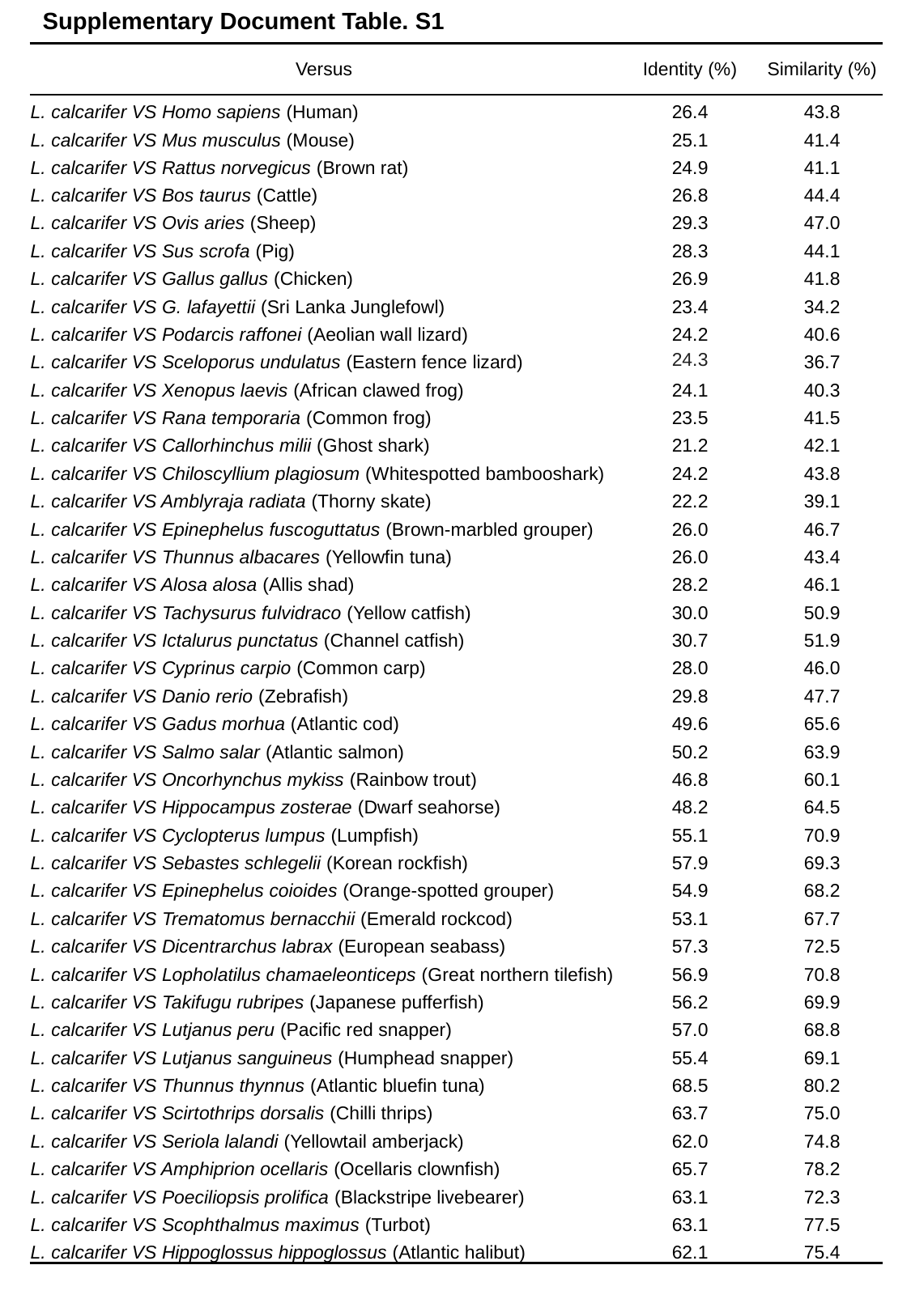

Supplementary Document Table. S1
| Versus | Identity (%) | Similarity (%) |
| --- | --- | --- |
| L. calcarifer VS Homo sapiens (Human) | 26.4 | 43.8 |
| L. calcarifer VS Mus musculus (Mouse) | 25.1 | 41.4 |
| L. calcarifer VS Rattus norvegicus (Brown rat) | 24.9 | 41.1 |
| L. calcarifer VS Bos taurus (Cattle) | 26.8 | 44.4 |
| L. calcarifer VS Ovis aries (Sheep) | 29.3 | 47.0 |
| L. calcarifer VS Sus scrofa (Pig) | 28.3 | 44.1 |
| L. calcarifer VS Gallus gallus (Chicken) | 26.9 | 41.8 |
| L. calcarifer VS G. lafayettii (Sri Lanka Junglefowl) | 23.4 | 34.2 |
| L. calcarifer VS Podarcis raffonei (Aeolian wall lizard) | 24.2 | 40.6 |
| L. calcarifer VS Sceloporus undulatus (Eastern fence lizard) | 24.3 | 36.7 |
| L. calcarifer VS Xenopus laevis (African clawed frog) | 24.1 | 40.3 |
| L. calcarifer VS Rana temporaria (Common frog) | 23.5 | 41.5 |
| L. calcarifer VS Callorhinchus milii (Ghost shark) | 21.2 | 42.1 |
| L. calcarifer VS Chiloscyllium plagiosum (Whitespotted bambooshark) | 24.2 | 43.8 |
| L. calcarifer VS Amblyraja radiata (Thorny skate) | 22.2 | 39.1 |
| L. calcarifer VS Epinephelus fuscoguttatus (Brown-marbled grouper) | 26.0 | 46.7 |
| L. calcarifer VS Thunnus albacares (Yellowfin tuna) | 26.0 | 43.4 |
| L. calcarifer VS Alosa alosa (Allis shad) | 28.2 | 46.1 |
| L. calcarifer VS Tachysurus fulvidraco (Yellow catfish) | 30.0 | 50.9 |
| L. calcarifer VS Ictalurus punctatus (Channel catfish) | 30.7 | 51.9 |
| L. calcarifer VS Cyprinus carpio (Common carp) | 28.0 | 46.0 |
| L. calcarifer VS Danio rerio (Zebrafish) | 29.8 | 47.7 |
| L. calcarifer VS Gadus morhua (Atlantic cod) | 49.6 | 65.6 |
| L. calcarifer VS Salmo salar (Atlantic salmon) | 50.2 | 63.9 |
| L. calcarifer VS Oncorhynchus mykiss (Rainbow trout) | 46.8 | 60.1 |
| L. calcarifer VS Hippocampus zosterae (Dwarf seahorse) | 48.2 | 64.5 |
| L. calcarifer VS Cyclopterus lumpus (Lumpfish) | 55.1 | 70.9 |
| L. calcarifer VS Sebastes schlegelii (Korean rockfish) | 57.9 | 69.3 |
| L. calcarifer VS Epinephelus coioides (Orange-spotted grouper) | 54.9 | 68.2 |
| L. calcarifer VS Trematomus bernacchii (Emerald rockcod) | 53.1 | 67.7 |
| L. calcarifer VS Dicentrarchus labrax (European seabass) | 57.3 | 72.5 |
| L. calcarifer VS Lopholatilus chamaeleonticeps (Great northern tilefish) | 56.9 | 70.8 |
| L. calcarifer VS Takifugu rubripes (Japanese pufferfish) | 56.2 | 69.9 |
| L. calcarifer VS Lutjanus peru (Pacific red snapper) | 57.0 | 68.8 |
| L. calcarifer VS Lutjanus sanguineus (Humphead snapper) | 55.4 | 69.1 |
| L. calcarifer VS Thunnus thynnus (Atlantic bluefin tuna) | 68.5 | 80.2 |
| L. calcarifer VS Scirtothrips dorsalis (Chilli thrips) | 63.7 | 75.0 |
| L. calcarifer VS Seriola lalandi (Yellowtail amberjack) | 62.0 | 74.8 |
| L. calcarifer VS Amphiprion ocellaris (Ocellaris clownfish) | 65.7 | 78.2 |
| L. calcarifer VS Poeciliopsis prolifica (Blackstripe livebearer) | 63.1 | 72.3 |
| L. calcarifer VS Scophthalmus maximus (Turbot) | 63.1 | 77.5 |
| L. calcarifer VS Hippoglossus hippoglossus (Atlantic halibut) | 62.1 | 75.4 |

## Slide 2
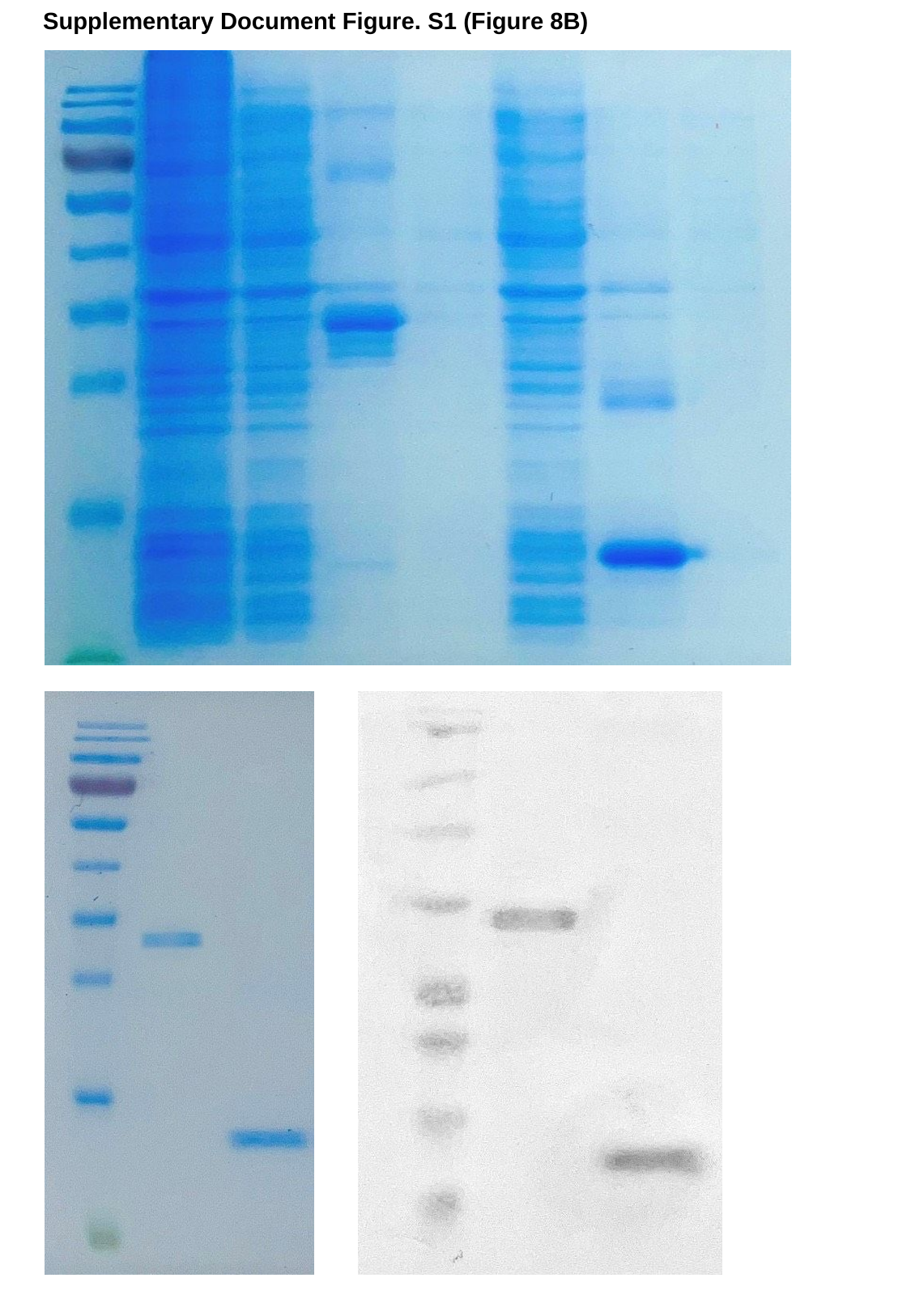

Supplementary Document Figure. S1 (Figure 8B)
